# Enhanced durability predicts success in amateur road cycling: evidence of power output declines

**DOI:** 10.3389/fspor.2025.1530162

**Published:** 2025-05-14

**Authors:** Artur Barsumyan, Christian Soost, Rene Burchard

**Affiliations:** ^1^ Faculty of Medicine, Philipps-University of Marburg, Marburg, Germany; ^2^ Department of Orthopedics and Trauma Surgery, Sports Medicine and Joint Centre, Lahn-Dill-Kliniken, Wetzlar/Dillenburg, Germany; ^3^ Faculty III: Statistic and Econometrics, University of Siegen, Siegen, Germany; ^4^ Department of Orthopedics and Traumatology, University Hospital of Giessen and Marburg, Marburg, Germany

**Keywords:** durability, resilience, amateur, cycling, remote coaching

## Abstract

**Purpose:**

Durability refers to an athlete's capacity to sustain optimal performance levels during prolonged physical exertion. Durability has been recognized as important and remains a key factor in endurance performance, particularly among amateur athletes who make up the largest segment of the endurance sports community. In the modern era, where remote coaching has become increasingly prevalent, there is a need for new methods to measure durability effectively without the constraints of laboratory. The aim of this study was to quantify durability in well-trained age-group cycling athletes using home-based test measures, identify durability as an important predictor of endurance performance, and provide practical recommendations for improving durability through training.

**Methods:**

Fourteen endurance-trained cyclists (mean 37.5 ± 5.7 years; VO_2_max 52.0 ± 7.4 ml·kg^−^¹·min^−^¹; training volume 9.6 ± 2.2 h·week^−^¹) took part in this study. Participants were divided into two groups based on less successful achievements: Power output and heart rate response for 5- and 20 min time trial (TT) efforts was measured in watt under both fresh and fatigued conditions. The fatiguing protocol involved cycling at 70%–80% of participants' initial 20 min TT power until 1,000 kJ of work was completed, followed by a 5- and 20 min TT.

**Results:**

Successful amateur cyclists have a significantly lower drop of power of in a 20 min interval in fatigued condition compared to less successful counterparts. The average drop in power is only half as high for successful athletes (6.5%) as it is for the less successful athletes (12.5%). For the 5 min interval and the heart rate response between fresh and fatigue state, no differences could be found.

**Conclusion:**

The findings of this study demonstrate that successful amateur cyclists exhibit better durability than less successful athletes after a defined amount of work, enabling them to sustain higher performance levels during prolonged efforts.

## 1 Introduction

Durability, often synonymous resilience, refers to an athlete's ability to maintain optimal performance over extended periods of physical exertion (1). In endurance sports, such as road cycling, durability is emerging as a critical determinant of success, especially during the later stages of events that demand sustained high outputs after significant energy expenditure. Unlike traditional performance metrics like VO_2_₂ max, lactate threshold, and critical power, which provide snapshots of an athlete's acute performance capacity, as well as skeletal muscle determinants of endurance performance, durability focuses on how performance declines over time due to factors such as muscle glycogen depletion, metabolite accumulation, and central nervous system fatigue (2–6).

The concept of durability has gained numbers of publications in recent years, offering explanations for the performance behaviors long observed by cyclists and coaches. This addresses why some athletes can produce high power outputs even after accumulating substantial workloads, often measured in kilojoules (7). Decisive moments in these races usually occur after several hours of riding, making an athlete's durability a key factor that distinguishes more successful cyclists from their less successful counterparts.

Durability has been recognized as important and remains a key factor in endurance performance, particularly among amateur athletes who make up the largest segment of the endurance sports community. Most existing research focuses on acute performance measures of professional athletes, failing to capture how performance declines over time among nonprofessional competitors (8). Measuring durability poses significant challenges owing to the need for longitudinal data that track physiological and mechanical parameters over extended durations, often during competitions. The complex interplay between training load, recovery, and fatigue further complicates the isolation of durability as a single variable.

In the modern era, where remote coaching has become increasingly prevalent (9), there is a pressing need for new tools, methods, and tests to effectively quantify, measure, and train durability. Coaches and athletes are seeking practical ways to assess durability without the constraints of laboratory settings or in-person evaluation. Developing standardized field tests that can simulate race conditions and quantify durability in terms of the work completed (e.g., kilojoules expended) would be highly valuable for training and performance optimization.

This study aimed to quantify the durability of competitive amateur cyclists by examining the decline in power output during pre-season time trials. Additionally, it sought to identify durability as a key predictor of endurance performance and provide practical insights into how durability can be measured and enhanced, offering evidence-based training strategies for coaches and athletes in remote settings.

As endurance sports continue to evolve and the demands on athletes to consistently perform at high levels over longer periods of time increase, understanding and improving their endurance is becoming increasingly important. By focusing on durability as a critical aspect of endurance performance, this study aimed to bridge the gap between physiological theory and practical applications. The results could inform training methodology and help athletes to outperform previous benchmarks and achieve greater success in road cycling.

## 2 Materials and methods

### 2.1 Study design

**Figure 1:**
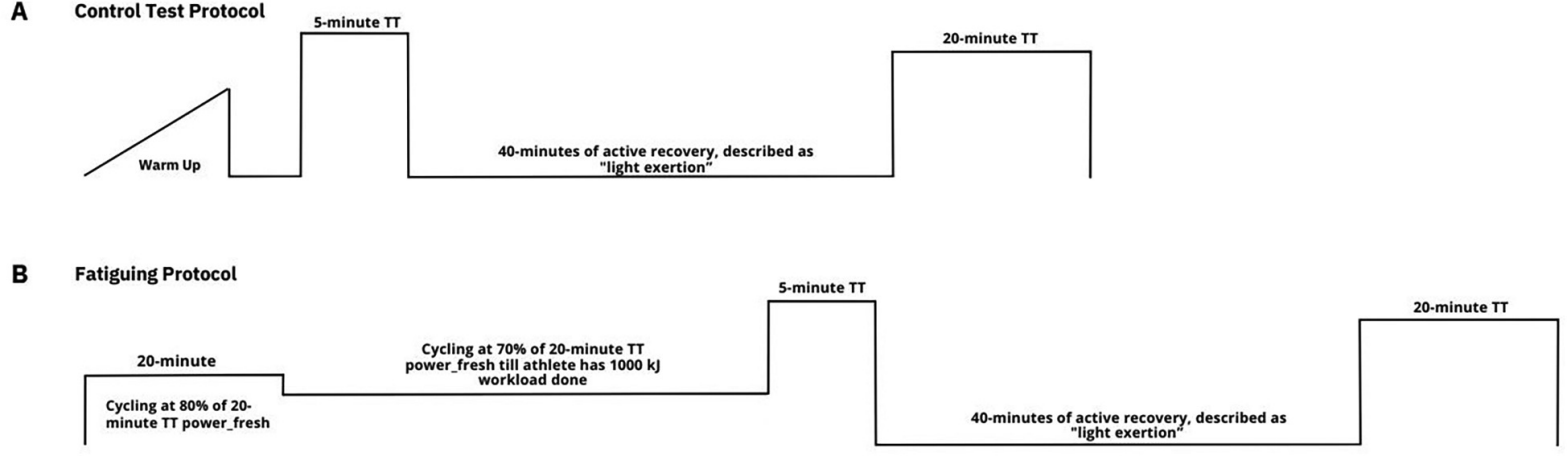
Schematic overview of the control test **(A)** and the fatiguing protocol **(B)**.

During the early season (April to May 2024), before athletes had participated in any races, participants completed four time trials (TT) spread over two weekends. The first two TT were conducted in a fresh state, whereas the subsequent two TTs were performed after a fatiguing protocol (see Figure 1), as detailed below.

### 2.2 Participants

Fourteen endurance-trained cyclists took part in the study. Body mass was measured before the first TT under fresh conditions. The VO_2_max data were obtained from the last standardized cardiopulmonary exercise test performed no longer than four months before the start of the study.

Inclusion criteria were as follows.

– Habitual training volume exceeding 7 h·week^−^¹.
– Free from viral infections and musculoskeletal injuries for at least three months prior to the study.
– Minimum of three years of experience in endurance sports.
– Coached by the same coach in the year preceding the tests.
– Regular participation in competitions ranging from domestic to international levels.
– No professional licenses held (nor a member of any UCI Continental, Pro or World Tour team).

The participants were divided into two groups:

1. Successful Group (*n* = 7): Athletes who achieved at least a podium finish in domestic-level competitions or qualified for UCI world championship road cycling in the previous season.
2. Less successful Group (*n* = 7): Athletes who regularly competed but did not attain the achievements of the first group.

This study was conducted in accordance with the Declaration of Helsinki (10) and the ethical board of the Philipps-University of Marburg approved the study (24-279 RS). Informed consent was obtained from all participants after verbal and written explanations of the experimental protocol and potential risks were provided. The study was not registered in a database. The data are available from the corresponding author upon reasonable request.

### 2.3 Control test protocol

Tests were conducted on two consecutive weekends. Participants were instructed to consume a standardized breakfast containing approximately 2 g·kg^−^¹ of carbohydrates and ∼800 ml of water one hour before testing, adhering to general pre-exercise nutritional guidelines (11). They were advised to avoid caffeine, vigorous exercise, and alcohol for 24 h prior to each test and to standardize their food intake in the evening before. During tests carbohydrate intake was standardized at 60 g·h^−^¹ using a 1:1 ratio of glucose and fructose provided through energy drinks and gels. The participants refrained from consuming caffeine during the fatiguing protocol and were allowed to drink water *ad libitum*.

After a 20 min standardized warm-up phase, the first test was carried out by means of 5 min and 20 min performance tests under fresh conditions [5′-Power output (PO)_fresh_ and 20′-PO_fresh_]. The second test, which was performed according to a fatigue protocol (see below), was used to derive the 5 min and 20 min performance under fatigue.

The 5 min and 20 min bouts of fresh and fatigued exercise were separated by 40 min of active recovery, during which participants maintained a predetermined subjective effort that did not exceed 2 out of 10 on Borg's 10-point scale, equivalent to a “light effort” (12). Participants were allowed to freely choose their cadence during the fatigue protocol. Before each effort, participants were encouraged to achieve a maximum workload while maintaining a cadence between 80 and 100 revolutions per minute. The power values of the 5 min and 20 min maximal effort TT were plotted against 1/time (t), where t is the corresponding duration, to linearize the relationship between power and duration (13).

### 2.4 Fatiguing protocol

Prior to the second set of 5 min and 20 min tests, participants completed a fatiguing protocol consisting of:

– 20 min at 70% of their initial 20′-PO_fresh_ as warm up.
– Continuous cycling at 80% of their initial 20′-PO_fresh_ until they expended 1,000 kJ of work. The total workload was calculated together with first initial 20 min warm up.
– Carry out a 5 min TT test immediately afterwards. (5′-PO_fatigue_).
– 40 min of active recovery after the 5 min TT test.
– Carry out a 20 min TT test immediately afterwards. (20′-PO_fatigue_).

### 2.5 Power output data

All tests were performed using the participants' own road bicycles mounted on direct-drive, electromagnetically braked indoor cycling trainers (Kickr v5, Wahoo Fitness, Atlanta, USA; Tacx, Tacx, Wassenaar, The Netherlands). These indoor trainers were calibrated according to the manufacturers' specifications to ensure valid and reliable power output measurements (14). All participants used fans for cooling and conducted the tests in a well-ventilated room. Power output was measured using two brands of power meters: Garmin Rally (Garmin Ltd., Olathe, Kansas, USA) and Assioma (Favero Electronics, Treviso, Italy), recorded on portable head units (Garmin Edge 520 or Garmin Edge 1,040, Garmin Ltd., Olathe, Kansas, USA). Participants performed a “zero offset” calibration before each test, following manufacturer guidelines. Data files with irregularities, such as missing or erroneous power readings (e.g., sudden spikes or drops in power output that were not associated with corresponding changes in cadence or heart rate, as well as prolonged zero values despite ongoing activity) or incomplete data due to technical issues (e.g., battery failure), were excluded from analysis. Power output data were analyzed and visually inspected for errors by two independent researchers using commercial software (WKO 5 build 590).

### 2.6 Heart rate data

Heart rate responses were assessed during both the 5 min and 20 min time trials to evaluate changes between fresh and fatigued states. Heart rate data were continuously recorded throughout each test using a heart rate chest strap (Garmin HRM-Pro, Garmin Ltd., Olathe, Kansas, USA), paired with portable head units. Comparisons were made between the fresh (first test) and fatigued (second test) conditions. These values were compared between successful and less successful athletes to determine whether fatigue influenced heart rate responses differently across groups.

### 2.7 Statistical analysis

The sample size was calculated *a priori* using G*Power (15). To achieve a statistical power of 80% at an alpha level of 0.05 and assuming a large effect size (Cohen's d = 0.4 for ANCOVA), the required total sample size of 26 participants was suggested. However, due to only 14 available athletes without exclusion criteria during the study period, the final sample comprised only 14 participants. The statistical analysis was performed using R (R Foundation for Statistical Computing, Vienna, Austria) on the basis of the available test subjects. Data are presented as mean ± standard deviation unless stated otherwise. The changes in power outputs were shown as a percentage. The Analysis of Covariance (ANCOVA) was used to analyze the differences in power output against the background of possible confounders. To fulfill the statistical requirements of ANCOVA the percentage changes were transformed into logits (16). *T*-tests were used for bivariate mean comparisons. Statistical significance was set at *p* < 0.05. Asterisks indicate statistical significance, with more asterisks representing lower *p*-values (e.g., *p < 0.05, **p < 0.01, *p < 0.001).

## 3 Results

### 3.1 Baseline physiological characteristics

The analysis of the socio-demographic characteristics of the successful and less successful athletes reveals significant differences in weight, VO_2_max and weekly training whereby age differences could not be determined (Table 1).

**Table 1:** Baseline characteristics such as body weight, training volume, maximal oxygen uptake and age pattern of successful and less successful amateur cyclists.

Variable	Groups of athletes		*p*-value
	Less successful	Successful	
Body weight (kg)	78.3 (8.2)	68.3 (6.8)	0.03*
VO_2_ max (ml·kg^−^¹·min^−^¹;)	47.4 (2.3)	56.6 (8.1)	0.02*
Weekly training volume (h)	8.2 (1.7)	11.1 (1.7)	0.01**
Age (years)	40.3 (3.9)	34.7 (6.2)	0.07

Values are mean sd, standard deviation; VO_2_ max, maximal oxygen uptake.

*p < 0.05.

**p < 0.01.

In the absence of age differences, the group of successful athletes consistently shows parameters that are conducive to higher performance. To rule out the possibility that the core results of this study can be explained solely by these parameters, a univariate model including the parameters shown in Table 1 was carried out for the comprehensive static analysis.

### 3.2 Changes in cycling performance and heart rate response in fresh and fatigue state between successful and less successful athletes

Table 2 shows the mean absolute changes of cycling performance and heart rate response and reveals that successful amateur cyclists have had a significantly smaller decrease in power output during the 20 min time trial under fatigue.

**Table 2:** Cycling performance (W; in watt; after the 5 min TT and after the 5 and the 20 min TT) and heart rate (HR; measured in beats per minute; 20 and 5 min after test finish) response in fresh and fatigue state between successful and less successful athletes.

Variable	Groups of athletes			
	Successful		Less successful	
	Fresh	Fatigue	Fresh	Fatigue
W 5 min	300.57 (55.47)	276.43 (46.03)	298.57 (43.35)	254.29 (41.65)
W 20 min	259.14 (44.29)	245.00 (45.78)	242.14 (24.46)	(204.57) (23.34)
HR 5 min	171.57 (6.92)	174.86 (6.96)	164.00 (10.74)	167.14 (10.04)
HR 20 min	166.14 (6.84)	169.00 (6.11)	155.14 (13.75)	158.86 (10.88)

Values are mean sd, standard deviation.

**Figure 2:**
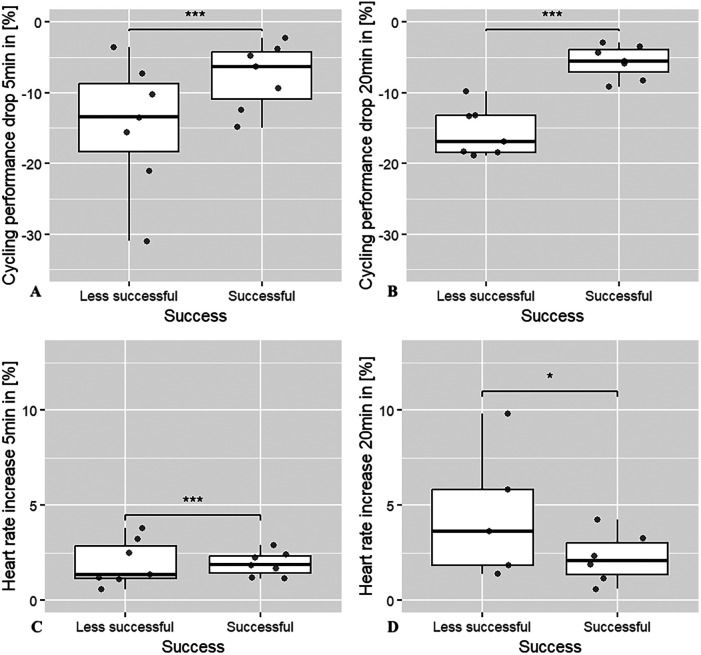
Boxplot comparison of power output drop and heart rate response, both in percentage values, in fresh and fatigue state between successful and less successful amateur cyclists during 5 min and 20 min intervals. **(A)** drop of power output in % from 5′- PO_fresh_ to 5′- PO_fatigue_. **(B)** drop of power output in % from 20′- PO_fresh_ to 20′- PO_fatigue_. **(C)** increase of heart rate in % from 5′- PO_fresh_ to 5′- PO_fatigue_. **(D)** increase of heart rate in % from 20′- PO_fresh_ to 20′- PO_fatigue_.

Figure 2 shows the mean absolute changes in cycling performance and heart rate response comparing the successful and less successful athletes in fresh and fatigued states. This figure with direct bivariate comparisons illustrates how the two groups differ in their ability to maintain performance under fatigue. However, due to the potential confounding effects of significantly different body weight, VO_2_max and weekly training volume, these differences need to be tested for validity in the following analysis of covariance.

The ANCOVA shows that successful amateur athletes have a significantly lower drop in performance in the 20 min interval (*p* = 0.033) than their less successful counterparts (see Table 3). Based on the adjusted mean values, a mean performance drop of 6.5% can be calculated for successful athletes and 12.5% for less successful ones, which corresponds to a performance drop that is twice as high for the less successful athletes. In contrast to this finding, no significant difference was found for the 5 min interval.

**Table 3:** ANCOVA results: power drop between successful and less successful athletes.

Variable	Data of W5 min drop				Data of W20 min drop			
	Sum Sq	Df	F	*p*	Sum Sq	Df	F	*p*
Intercept	1.7166	1	2.6743	0.1364	0.08486	1	1.0108	0.34096
Successful vs. Less successful	0.4754	1	0.7406	0.4118	0.53047	1	6.3187	0.03311*
VO_2_max	1.0224	1	1.5928	0.2387	0.79845	1	9.5107	0.01306*
Training	0.4161	1	0.6483	0.4415	0.06411	1	0.7636	0.40491
Weight	0.2440	1	0.3801	0.5528	0.01519	1	0.1809	0.68057
Residuals	5.7769	9			0.75557	9		

*p < 0.05.

**Figure 3:**
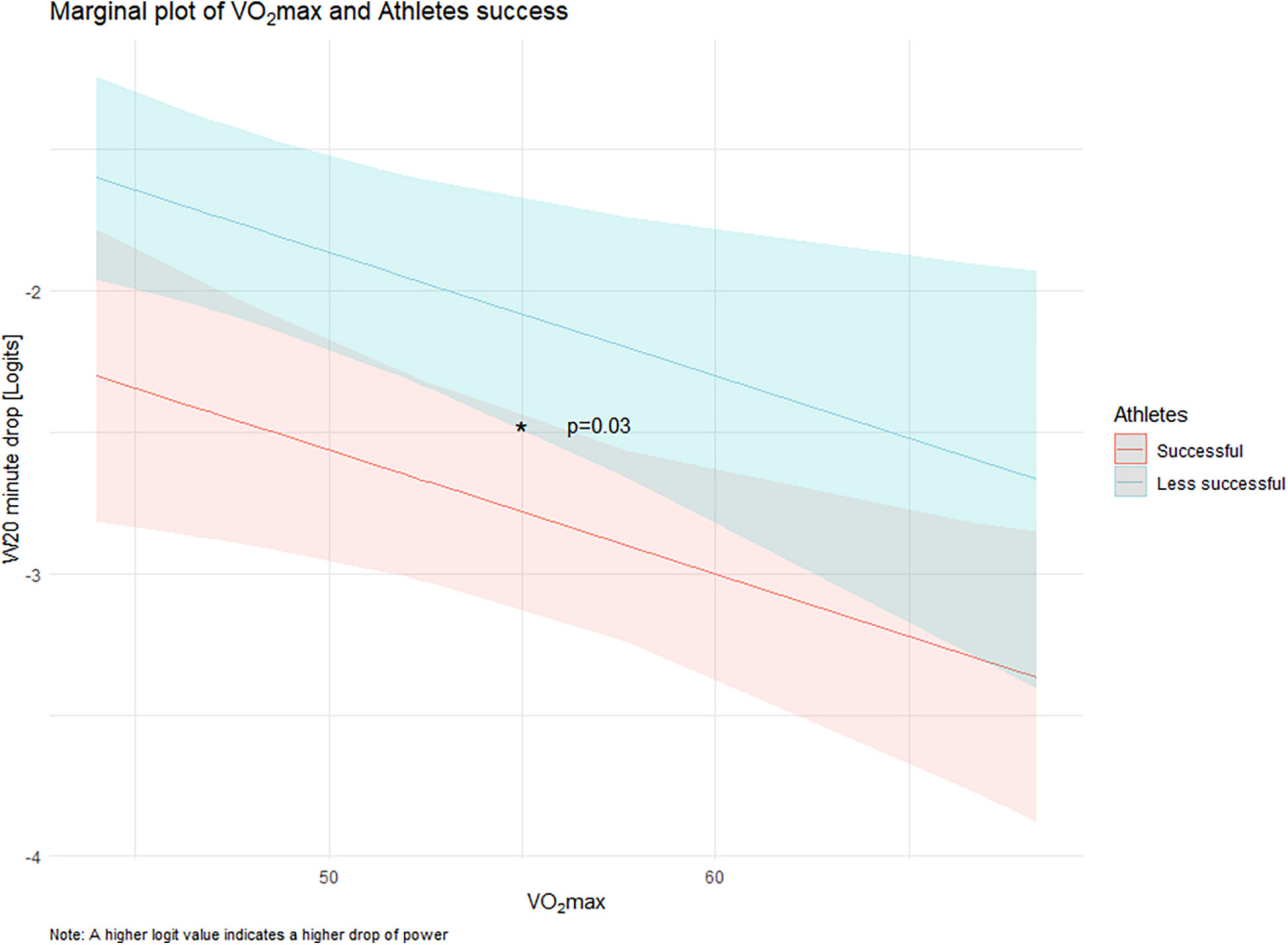
Comparison of power output decline in fresh and fatigue state between successful and less successful amateur cyclists during 5 min and 20 min intervals.

Figure 3 shows the marginal plot of VO_2_max and 20 min power decrement, comparing successful and less successful amateur athletes. The *y*-axis represents the drop in power over 20 min, expressed in logit transformed values, where higher logit values correspond to a greater drop in performance and the *x*-axis shows VO_2_max.

The plot shows a significant interaction (*p* = 0.03), indicating that less successful athletes (blue line) show a steeper decline in performance as VO_2_max decreases compared to successful athletes (red line). The shaded areas represent the 95% confidence intervals and illustrate the variability within each group.

**Figure 4:**
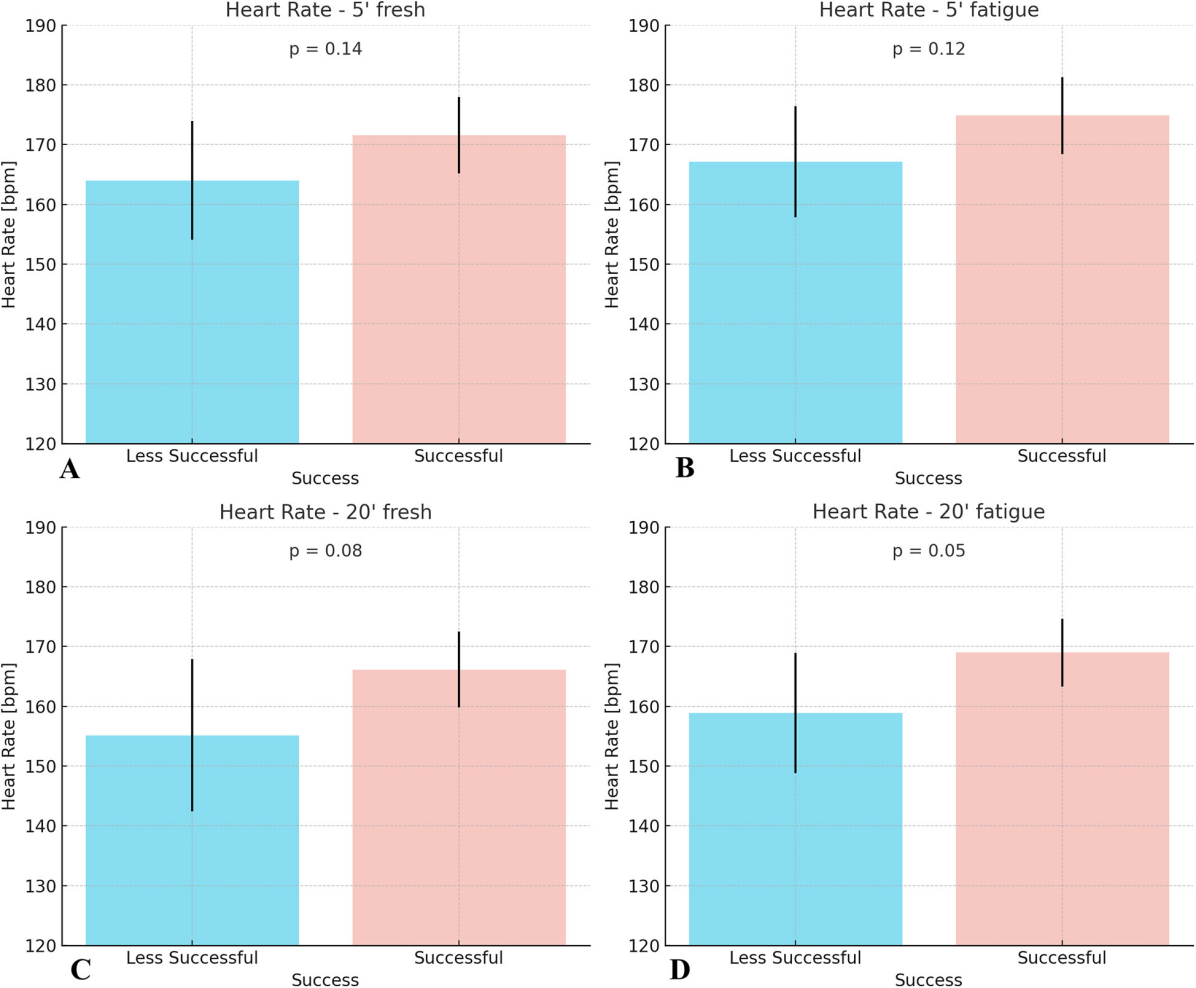
Mean heart rate responses of less successful and successful amateur cyclists during 5 min and 20 min time trials performed under fresh and fatigued conditions. **(A)** heart rate at 5 min under fresh condition; **(B)** heart rate at 5 min under fatigued condition; **(C)** heart rate at 20 min under fresh condition; **(D)** heart rate at 20 min under fatigued condition.

Figure 4 represents the mean heart rate in beats per minute (bpm), and error bars indicate ± standard deviation. There were no significant differences in heart rate response between the two study groups (Table 4).

**Table 4:** ANCOVA results heart rate response.

Variable	Data of heart rate response 5 min				Data of heart rate response 20 min			
	Sum Sq	Df	F	*p*	Sum Sq	Df	F	*p*
Intercept	0.9835	1	2.5046	0.1480	0.2436	1	0.4254	0.5306
Successful vs. Less successful	0.0061	1	0.0156	0.9034	0.0244	1	0.0426	0.8411
VO_2_max	0.0142	1	0.0361	0.8535	0.9421	1	1.6450	0.2317
Training	0.0135	1	0.0345	0.8568	0.0242	1	0.0422	0.8419
Weight	0.0068	1	0.0174	0.8980	0.0202	1	0.0353	0.8552
Residuals	3.5339	9			5.1542	9		

## 4 Discussion

Recent studies have highlighted the importance of durability in endurance performance. Maunder et al. (17) emphasized that traditional methods of assessing endurance often overlook how physiological variables deteriorate over time during prolonged exercise. They defined durability as “the time of onset and magnitude of deterioration in physiological characteristics over time during prolonged exercise”. This view, like ours, suggests that ignoring durability can lead to an incomplete understanding of an athlete's performance.

Studies with professional cyclists have shown that durability differentiates more successful cyclists from their less successful counterparts. For example, Leo et al. (18) analyzed performance data from professional and U23 cyclists during the Tour of the Alps and found that professional cyclists maintained a higher relative power output in the later stages of the race when compared to their less experienced and younger counterparts. Similarly, van Erp et al. (8) reported that successful climbers and sprinters showed a lower power drop after high amounts of work compared to less successful riders. Our study demonstrated comparable findings, indicating that successful riders exhibit greater durability, as evidenced by a lower decline in power output following high workloads.

The study presented here extends these findings to the level of amateur cyclists. To our knowledge, limiting numbers of study has directly quantified the relationship between durability and performance outcomes in amateur cyclists. Only Stevenson et al. (19) investigated the effects of prolonged cycling on the transition between low and moderate intensity exercise around first ventilatory threshold (VT_1_), based on data from amateur cyclists. Therefore, our findings provide novel insights into how durability plays a crucial role in amateur endurance athletes, similar to what has been demonstrated in professional cycling (8).

These findings highlight the importance of individualized training programs that consider changes in physiological responses over longer periods of time. For amateur athletes, maintaining a constant power output in the upper range of low intensity could lead to an unintentional shift to moderate intensity as fatigue sets in. The development of durability appears to be closely linked to long-term training consistency, high training volumes, and the inclusion of prolonged exercise bouts incorporating high-intensity efforts or progressive intensity increases (20).

Monitoring heart rate response and adjusting exercise intensity could therefore help to control fatigue and improve endurance (21). Understanding the mechanisms underlying the decline in performance during prolonged exercise is crucial for the development of effective training strategies to improve durability. But we found no significant difference in heart rate response between two groups of athletes. However, during prolonged exercise, heart rate can progressively increase, even when the power output remains constant (22). This demonstrates that an athlete using heart rate to regulate training intensity may unintentionally reduce their power output over time to maintain their target heart rate zone. Interestingly, the extent to which cardiovascular response during prolonged exercise reflects changes in intensity transitions is not yet fully understood. However, our finding underscores the limitations due the absence of standardized procedures to account for changes in physiological profiling variables during prolonged exercise. As a result, it remains unclear whether the decoupling between heart rate and power represents shifts in exercise intensity domains or is merely a harmless response that doesn't require adjustment when controlling training load. Gaining insight into the relationship between cardiovascular response and the immediate, exercise-induced changes in power output at these intensity transitions has important implications for how heart rate monitoring can be effectively utilized for regulating training intensity during prolonged exercise sessions.

Unfortunately, most studies on durability have been conducted with small numbers of subjects—9 rides was in both studies Leo et al. (23), 10 athletes in the study by Spragg et al. (7), as was the case in our study (14 participants). Although there are currently no standardized field tests for assessing durability, each group of scientists has developed and used their own test (7, 19, 24). Our study presented contributes to this discourse by demonstrating that, among amateur athletes, durability can be assessed using a simple, remote testing protocol that does not rely on laboratory measurements. The developed test considers both the total work completed (in kilojoules) and the intensity of that work, aligning with suggestions from Leo et al. (25) that both factors are crucial for evaluating durability. By standardizing factors such as work intensity, nutritional intake, and recovery periods, our protocol allows for consistent and individualized assessment of durability in a home or remote setting. For well-trained cyclists, effective fatigue protocols often involve completing over 1,500 kJ or even 2,000 kJ of work, combined with high-intensity efforts above 80% of maximum heart rate. Coaches can conduct experiments with developing their own durability test protocols. To ensure accurate and comparable results, it is important to standardize key variables such as total work done (in kJ), the route, and energy intake before and during the test.

The primary outcome of this study was that alterations in the power-duration curve resulting from fatigue can be anticipated based on remote measurement data collected. These findings provide an opportunity for coaches to remotely assess durability, determine athlete profiling, and optimize training programming, offering crucial insights into how to effectively adjust training plans.

### 4.1 Practical applications

The most important practical finding from this study is that more successful amateur cyclists demonstrate greater durability, experiencing a smaller drop in performance over time compared to their less successful counterparts. This reinforces the crucial role of durability in endurance performance, particularly after substantial energy expenditure.

While the specific trainability of durability remains an area for further research, existing evidence suggests that its development is closely linked to long-term training consistency, high training volumes, and the inclusion of prolonged exercise sessions incorporating high-intensity efforts or progressive intensity increases. Coaches and athletes can apply these insights by integrating race-pace efforts late in training sessions, employing nutritional strategies to delay glycogen depletion, and monitoring heart rate and perceived exertion to manage fatigue effectively. Additionally, the ability to sustain power output under fatigue may serve as an indicator of an athlete's potential for endurance performance. This information can aid in talent identification and help guide training strategies tailored to improving resilience.

### 4.2 Limitations

Since the *a priori* power analysis, assuming a large effect size (Cohen's d = 0.4), suggested a larger number of subjects (*n* = 26) than was available for the study presented here, the size of the study population must be discussed as a major limiting factor. However, van Erp and colleagues (8), have been able to show validity of small study populations in this still young field of research about durability in cycling. For this reason, this study can be seen as a valuable contribution to the scientific world in the field of sports medicine and a corresponding template for further studies with a larger sample.

Moreover, the use of different brands of power meters to collect power data, which can lead to variations in measurements (26). Because study participants were not continuously observed before the testing sessions including the periods in between, we cannot completely guarantee the absence of protocol violations regarding adherence to the personal pre-test instructions. While our study did not assess cardiovascular drift in the conventional sense, we examined heart rate responses in 5- and 20 min time trials to evaluate transient changes between fresh and fatigued states, which may provide insights into short-duration submaximal effort-induced heart rate dynamics. Future studies with larger sample sizes of amateur athletes should incorporate analysis of the relationship between VO_2_max and durability to better quantify the variance explained by VO₂max. Another limitation is that most of the data was collected from male athletes. It remains unclear whether there is a similarly strong relationship between endurance and race performance in female cyclists, and further research is needed to investigate this aspect.

## Data Availability

The raw data supporting the conclusions of this article will be made available by the authors, without undue reservation.
